# Towards Global QSAR Model Building for Acute Toxicity: Munro Database Case Study

**DOI:** 10.3390/ijms151018162

**Published:** 2014-10-09

**Authors:** Swapnil Chavan, Ian A. Nicholls, Björn C. G. Karlsson, Annika M. Rosengren, Davide Ballabio, Viviana Consonni, Roberto Todeschini

**Affiliations:** 1 Bioorganic & Biophysical Chemistry Laboratory, Linnaeus University Centre for Biomaterials Chemistry and Department of Chemistry & Biomedical Sciences, Linnaeus University, Kalmar SE-391 82, Sweden; E-Mails: bjorn.karlsson@lnu.se (B.C.G.K.); annika.rosengren@lnu.se (A.M.R.); 2 Department of Chemistry-BMC, Uppsala University, Box 576, Uppsala SE-751 23, Sweden; 3 Milano Chemometrics and QSAR Research Group, Department of Earth and Environmental Sciences, University of Milano-Bicocca, Milano IT-20126, Italy; E-Mails: davide.ballabio@unimib.it (D.B.); viviana.consonni@unimib.it (V.C.); roberto.todeschini@unimib.it (R.T.)

**Keywords:** *k*-nearest neighbor (*k*-NN), Munro database, genetic algorithm (GA), acute toxicity (LD_50_)

## Abstract

A series of 436 Munro database chemicals were studied with respect to their corresponding experimental LD_50_ values to investigate the possibility of establishing a global QSAR model for acute toxicity. Dragon molecular descriptors were used for the QSAR model development and genetic algorithms were used to select descriptors better correlated with toxicity data. Toxic values were discretized in a qualitative class on the basis of the Globally Harmonized Scheme: the 436 chemicals were divided into 3 classes based on their experimental LD_50_ values: highly toxic, intermediate toxic and low to non-toxic. The *k*-nearest neighbor (*k*-NN) classification method was calibrated on 25 molecular descriptors and gave a non-error rate (NER) equal to 0.66 and 0.57 for internal and external prediction sets, respectively. Even if the classification performances are not optimal, the subsequent analysis of the selected descriptors and their relationship with toxicity levels constitute a step towards the development of a global QSAR model for acute toxicity.

## 1. Introduction

The Munro database is comprised of 613 chemicals representing a variety of pharmaceuticals, agricultural and industrial chemicals, substances used in food production and chemicals that have an impact on the environment [1]. A range of computational approaches has previously been developed for classifying the Munro database chemicals. The first effort was made by Munro *et al.*, in 1996, where classification was based upon types of chemical structure. The authors proposed the approach as a method for establishing a toxicological threshold of concern (TTC) for all Munro database chemicals. The authors used a decision tree approach [2] to classify the selected Munro database chemicals into one of the three structural classes and reported that the cumulative distributions of No Observe Effect Levels (NOELs) belonging to all chemicals varied considerably among all the three structural classes, which implied that “chemical structure defines toxicity”.

A scientific report submitted to European Food Safety Authority (EFSA) by Stocchero *et al.* [3] later demonstrated the strength of integrating physico-chemical data and toxicity data, in order to improve investigation of applicability of TTC schemes. The Principal Component Analysis (PCA) [4], Orthogonal Bidirectional Projections to Latent Structures-Discriminant Analysis (O2PLS-DA) [5] and clustering studies were carried out on Munro database chemicals using NOEL values as response variable. Applying these methods, the chemicals included in each study were initially divided into datasets based upon classes of hazard (I, II and III) and the results obtained were compared with data obtained after following a Cramer classification scheme [6]. Results confirmed that the Munro database is broadly representative of the chemical landscape, and the Cramer scheme could be robustly established for the classification of the database and emphasized the potential of chemoinformatics approaches for exploring relationships between chemical structure and toxicity.

Both the above mentioned studies incorporated sub-chronic toxicity endpoint data (NOEL) for classification of the Munro database while there are no published studies using acute toxicity endpoint data (like LD_50_, LC_50_, TD_50_) on the same database. The main benefit of acute toxicity values is that they are obtained in less time (1–4 days), which can contribute to cost efficiency, and with less cumbersome experiments as compared to those used for sub-chronic and chronic toxicity values, which generally take from 28 days to 2 years of study, involve huge amount of money and require significant effort. Moreover, in 1959, Russell and Burch established the 3Rs principle (replacement, reduction, and refinement) for animal research [7]. The REACH (Registration, Evaluation, Authorisation and Restriction of Chemical Substances) Article 25 (1) has clearly stated that unnecessary animal testing should be avoided and should only be undertaken as a last resort [8].

Thus, in this computational study we elected to substitute sub-chronic toxicity values (NOEL) of Munro database chemicals by acute toxicity values (LD_50_) prior to model development. These LD_50_ values were used to form classes of chemicals employing the Globally Harmonized Scheme (GHS) [9]. Accordingly, the aim of our research was to investigate whether it was possible to develop a physico-chemical parameter-based global model for acute toxicity through the application of the GHS for classifying Munro database chemicals by means of QSAR modelling.

## 2. Material and Methods

The Munro dataset was initially screened to ensure its consistency. The 613 chemicals included on the Munro database were examined and authenticated based on the correct structure, the correct IUPAC name and the correct CAS registry number (RN). The web-servers ChemSpider [10] and Cactus [11] were used to retrieve the IUPAC name and CAS number and match the information obtained from both webservers using InChI key as identifier. Salts and mixtures were removed from the original dataset. To calculate Dragon descriptors exact smile notation was used as input, and smiles notation for each structure were carefully checked in ChemSpider, SigmaAldrich [12] and PubChem [13]. The smiles for cis/trans isomers and R/S enantiomers were carefully inspected and only canonical smiles were taken into consideration.

All chemical structures containing diazo or guanidine functionalities were removed because of the presence of resonance structures, duplicate records and records missing either structure or CAS registry number were removed. There were 469 records that were found to have correct CAS, RN, IUPAC name and smile notation. The LD_50_ values (organism-rat, route-oral) for all those sorted records were searched for in Toxnet and RTECS webservers. Records with more than one endpoint value were removed. In the end, LD_50_ values for 441 chemicals (out of 469) were retrieved from Toxnet and RTECS.

Two-dimensional Dragon molecular descriptors were employed for model development. Three-dimensional descriptors were not calculated, since geometry optimization can be a time consuming step and consequently limit the future application of the proposed model. A total of 3668 descriptors were calculated for all 441 chemicals using Dragon 6 software [14]. The number of descriptors calculated for each Dragon block is shown in Table 1.

A filtering of the descriptors was performed in Dragon before exporting the descriptor values. Descriptors with one or more missing values were discarded, as well as constant, near constant and correlated descriptors. In the latter case, for each pair of descriptors with a correlation coefficient higher than 95%, the one showing the largest pair correlation with all the other descriptors was excluded. The reduced pool for the subsequent classification modeling included 1106 descriptors.

**Table 1:** Calculated 2D descriptors for 441 Munro database chemicals.

Sr.	Descriptor Type	No. of Descriptors
1	Constitutional indices	43
2	Topological indices	75
3	Connectivity indices	37
4	2D matrix based descriptors	550
5	ETA indices	23
6	Atom type E-state indices	170
7	2D atom pairs	1596
8	Drug like indices	27
9	Ring descriptors	32
10	Walk and path counts	46
11	Information indices	48
12	2D auto correlations	213
13	P-VSA like descriptors	45
14	Edge adjacency indices	324
15	CATS 2D	150
16	Atom-centered fragments	115
17	Molecular properties	20
18	Functional group counts	154
Total	All 18 types	3668

### 2.1. Descriptor Filtering and Outlier Detection

Principal Component Analysis (PCA) was used to initially filter molecular descriptors and, in particular, remove irrelevant ones. PCA is a multivariate technique that aims to reduce dimensional space of data by projecting it in the form of principal components. The largest variance is associated with first principal component and second largest with next principal component [4,15]. PCA was performed on all 441 chemicals using 1106 descriptors and ten principal components were considered (explained variance maximum of 20% with PC1 and minimum of 3% with PC10). The data was auto scaled prior to PCA analysis. Loading scores for all ten components were used as criteria to sort descriptors: 460 descriptors were found to have their loading values higher than a defined threshold (0.06), thus retained for further studies. Other descriptors were discarded, since they did not encode relevant information for the structural and chemical description of the dataset. Moreover, five chemicals were identified as potential outliers in PCA score plot (supplementary file). Thus 436 chemicals and 460 descriptors were finally retained for the subsequent model development.

### 2.2. Modelling Methods

#### 2.2.1. Classification Scheme

The quantitative toxicological response was discretized in a qualitative class on the basis of the GHS (Globally Harmonized Scheme).

Class I: LD_50_ ≤ 300 mg/kg/day;
Class II: 300 < LD_50_ ≤ 2000 mg/kg/day;
Class III: LD_50_ > 2000 mg/kg/day;

where, class I is the highly toxic, class II is the intermediate toxic and class III is the low to non-toxic class.

#### 2.2.2. *k*-Nearest Neighbors

The *k*-NN classification method was applied in order to find the appropriate relationship between molecular structures, encoded in molecular descriptors, and the toxicity of chemicals [16,17]. The *k*-NN classification rule is conceptually quite simple: a molecule is classified according to the classes of the *k* closest molecules, which means, it is classified according to the majority of its *k* nearest neighbors in the descriptors space. In this work, the Euclidean metric was used to measure distances between molecules. The *k* value giving the lowest classification error in cross-validation was selected as the optimal one.

#### 2.2.3. Descriptor Selection by Means of Genetic Algorithms

Genetic algorithms (GAs) efficiently perform global searches within a high-dimensional space and can remove variables which are non-significant for the modelled property, noisy or correlated by chance. GAs start from an initial random population of chromosomes, which are binary vectors representing the presence or absence of molecular descriptors. An evolutionary process is simulated to optimize a defined fitness function and new chromosomes are obtained by coupling the chromosomes of the initial population with genetic operations (crossover and mutation). To decide the number of evaluations a series of 40 runs were performed. The first 20 runs were performed using the original descriptors, while the next 20 runs were performed using randomly shuffled chemicals [18]. These two sets of 40 runs were compared to identify major differences in outcomes. The major difference was observed after 100 evaluations run and was used as the stopping criteria, *i.e.*, GA were performed with 100 evaluations. GA were optimized on the basis of the non-error rate (NER) which indicates the tendency of the model to correctly classify chemicals [19].

To retrieve all 1106 descriptors for all 436 chemicals, to perform PCA and to carry out descriptor selection by Genetic Algorithm; we have used “ga_toolbox” and “pca” Matlab modules developed at Milano Chemometrics and QSAR Research Group, University of Milano-Bicocca, Milano, Italy.

### 2.3. Model Validation

The 436 Munro dataset chemicals were divided into training and test sets. The chemicals were randomly split, keeping 80% of chemicals from every class in the training set and the remaining 20% in the test set, thus the selection was performed maintaining the class proportions. The training set was used to select molecular descriptors and to build the classification models. Molecules of the test set were used just to evaluate the predictive ability of the trained models. The distribution of the 436 chemicals into training and test sets is shown in Table 2.

**Table 2:** The distribution of class I, II and III chemicals into training and test sets based on the principle of keeping 20% of chemicals from each class as a test set.

	Class I	Class II	Class III	Total
Training	82	136	129	347
Test	21	35	33	89
Total	103	171	162	436

The training set of 347 chemicals with 460 descriptors was subjected to variable selection (by GA) coupled with *k*-NN classification, while the test set did not participate to the model calibration and was used to validate the model. The internal validation of models was assessed by 5-fold procedure. The 347 chemicals from the training set were divided in five groups and the prediction of class parameters of every fifth group were carried out using the remaining four groups. The class of test group chemicals was predicted based on classes of its *k* neighbors from training group. The best model, built on the training set that had the highest NER_cv_ and lowest class error, was subjected for external validation. The classification model’s performance was assessed by means of classification such as non-error rate (NER), sensitivity, specificity, precision and error rate (ER) [20]. All models were compared and the model with the lowest percentage error was chosen.

To classify chemicals into training and test sets as well as to perform GA-coupled *k*-NN classification the “classification toolbox” Matlab modules developed at Milano Chemometrics and QSAR Research Group were used [21]. The Matlab classification toolbox module is freely available online [22].

## 3. Results and Discussion

### 3.1. Genetic Algorithm

The descriptor selection based on the GA strategy was applied to all 460 descriptors to build a *k*-NN classification model using the three classes as response variable. As our objective was the development of a physico-chemical parameter-based model for the prediction of acute toxicity, ultimately for use in the rapid screening of compounds, we limited ourselves to the use of 2D descriptors. The best *k*-NN model found by means of GAs comprised 25 molecular descriptors and was associated to the NER_cv_ equals to 0.67 and NER_fit_ on the training set (fitting) equal to 0.66 (see Table 4). The “*k*” selection with 5-fold cross validation gave an optimal *k* value of 1. This means that just the closest molecule was used to calculate the class of each target molecule to be predicted. The 25 descriptors selected for *k*-NN classification are listed in Table 3.

The model was able to correctly classify 194 of 347 of the training set chemicals. The sensitivity describes the model’s ability to correctly identify the correct class for an object, here a chemical. In case of training set prediction, the *k*-NN classification cross-validated model shows sensitivities of 0.54, 0.49 and 0.65 for classes I, II and III respectively (Table 4). This statistic indicates that the *k*-NN classification model had 54% success in predicting highly toxic chemicals (class I), 49% for chemicals with intermediate toxicity (class II) and 65% for chemicals with low toxicity (class III). Specificity characterizes the ability of the particular class to reject molecules of all other classes. The *k*-NN classification cross-validated model shows high specificity values for all 3 classes. This indicates that the model can predict highly toxic chemicals (class-I) with a specificity rate of 0.81, 0.76 and 0.78 for classes II and III, respectively. When looking at the external validation set, the model demonstrated sensitivities of 0.39, 0.35 and 0.55 for classes I, II and III, respectively, and corresponding specificities of 0.73, 0.68 and 0.74. The model could correctly classify 38 of 89 of the external set chemicals. However, the slightly different performance between the training and test set was somehow expected, since test chemicals were not used in the model calibration. Importantly, our model data presented here can be compared with previous models correlating molecular structure and LD50 (organism-rat, route-oral) which yielded a predictive power associated with a r2 of less than 0.45 [23,24]. Keeping this in mind, our classification model did not show optimal performances in terms of a clear separation of toxicological classes, but did allow the establishment of a relationship between the molecular structures of chemicals included in the Munro dataset and their oral dosed acute toxicities. For this reason, molecular descriptors included in the model were further analyzed in order to better understand their role in determining the toxicity level of chemicals.

**Table 3:** Description of the 25 descriptors derived by the genetic algorithm coupled with *k*-NN classification.

Sr.	Name	Description	Type
1	MATS1e	Moran autocorrelation of lag 1 weighted by Sanderson electronegativity	2D autocorrelations
2	SpMAD_B(s)	Spectral mean absolute deviation from Burden matrix weighted by I-State	2D matrix-based descriptors
3	SpPosA_B(p)	Normalized spectral positive sum from Burden matrix weighted by polarizability	2D matrix-based descriptors
4	MATS1v	Moran autocorrelation of lag 1 weighted by van der Waals volume	2D autocorrelations
5	Mi	Mean first ionization potential (scaled on Carbon atom)	Constitutional indices
6	AAC	Mean information index on atomic composition	Information indices
7	SpMAD_B(m)	Spectral mean absolute deviation from Burden matrix weighted by mass	2D matrix-based descriptors
8	GATS1p	Geary autocorrelation of lag 1 weighted by polarizability	2D autocorrelations
9	C-026	R--CX--R	Atom-centred fragments
10	SIC0	Structural Information Content index (neighborhood symmetry of 0-order)	Information indices
11	nDB	Number of double bonds	Constitutional indices
12	SIC1	Structural Information Content index (neighborhood symmetry of 1-order)	Information indices
13	ATS6e	Broto-Moreau autocorrelation of lag 6 (log function) weighted by Sanderson electronegativity	2D autocorrelations
14	P_VSA_MR_3	P_VSA-like on Molar Refractivity, bin 3	P_VSA-like descriptors
15	DLS_02	Modified drug-like score from Oprea *et al.*, (6 rules)	Drug-like indices
16	nCL	Number of Chlorine atoms	Constitutional indices
17	J_Dz(Z)	Balaban-like index from Barysz matrix weighted by atomic number	2D matrix-based descriptors
18	SM6_B(s)	Spectral moment of order 6 from Burden matrix weighted by I-State	2D matrix-based descriptors
19	GATS1v	Geary autocorrelation of lag 1 weighted by van der Waals volume	2D autocorrelations
20	JGI4	Mean topological charge index of order 4	2D autocorrelations
21	P_VSA_i_4	P_VSA-like on ionization potential, bin 4	P_VSA-like descriptors
22	P-117	X3-P = X (phosphate)	Atom-centred fragments
23	B01[S-P]	Presence/absence of S–P at topological distance 1	2D Atom Pairs
24	B03[C-S]	Presence/absence of C–S at topological distance 3	2D Atom Pairs
25	BLTF96	Verhaar Fish base-line toxicity from MLOGP (mmol/L)	Molecular properties

**Table 4:** Classification parameters of *k*-NN classification model.

	NER	ER	Sensitivity			Specificity		
			Class			Class		
			I	II	III	I	II	III
Fitting	0.66	0.34	0.53	0.46	0.65	0.80	0.74	0.78
cv	0.67	0.33	0.54	0.49	0.65	0.81	0.76	0.78
External	0.57	0.43	0.39	0.35	0.55	0.73	0.68	0.74

### 3.2. Analysis Based on 25 Descriptors

To determine how structures were related with toxicity classes, we performed a Principal Component Analysis (PCA) study on both training set and test set chemicals using the 25 descriptors selected for the *k*-NN classification model.

#### 3.2.1. Score Plot

**Figure 1:**
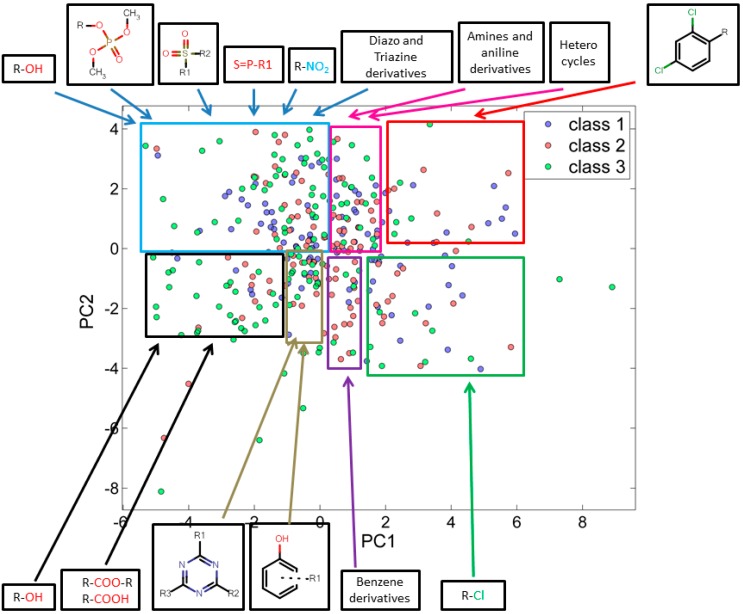
PCA score plot using 25 descriptors explaining similarity and variability in training set chemicals with respect to their corresponding class.

In the score plot, similar chemicals lie closer to each other with respect to the first two principal components. Whereas the chemicals which differ from each other are found further away from each other. Figure 1 illustrates how chemicals are distributed based on their similarities and differences, all chemicals are denoted by three different colors with their respective class (*i.e*., I (blue), II (red) and III (green)). PC-1 has shown 20% of variance while PC-2 has shown 16% of variance. For the 347 training set chemicals the total variance associated with first two components was found to be 36%. PCA correctly identified a molecular clustering based on the structural properties of molecules. By comparing score and loading plots, it is clear how clusters in the score plot are characterized by molecules with similar structures, as well as how the selected molecular descriptors encode the correct information to visualize and separate these structural clusters.

In fact, most of the aliphatic alcohols were present at the left most side of the score plot, while halogenated benzenes were clustered on the right most side along principal component-1 (PC-1). The third distinct functional group was the halogenated alkenes and halogenated heterocyclic structures, which were projecting downward along with the PC-2. Those chemicals containing phosphate and sulphate functionalities were found to increase in the upward direction along with PC-2. A series of distinct characteristic structural sets are shown grouped with marked areas in Figure 1.

**Figure 2:**
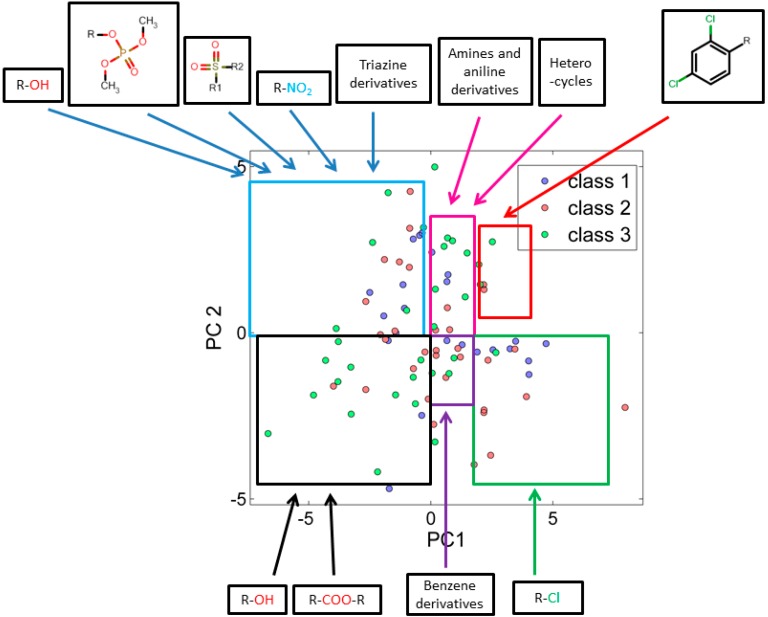
PCA score plot using 25 descriptors explains similarity and variability in test set chemicals with respect to their corresponding class.

When score plots of test set (Figure 2) was compared with training set (Figure 1), we found that the many similar functional groups formed clusters.

Analysis of the score plots for training and test set chemicals showed that the classification model revealed a number of trends, the red and green marked sectors of Figure 1 and Figure 2 have a slightly higher frequency of highly toxic chemicals (class-I) and a region (purple marked sectors) dominated by intermediate toxic chemicals (class-II). The blue, magenta, black and grey marked areas reveal on the contrary a higher frequency of class-III chemicals, especially in the most remote areas of the clusters. This can suggest a potential and underlying trend of relationships between the first PC and the toxicity level, which should be better clarified and analyzed in future studies.

#### 3.2.2. Loading Plot

**Figure 3:**
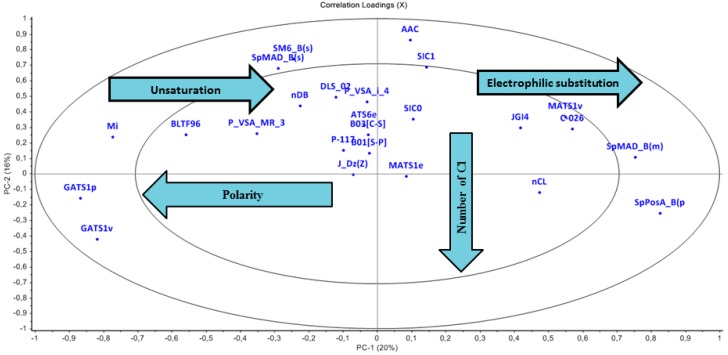
Loading plot describes significant descriptors.

The PCA Loading plot (Figure 3) was used as to analyse the importance of molecular descriptors for determining each component and thus the role of descriptors for the separation of clusters identified in the score plot. Chemicals placed in the left side of the score plot (with negative scores on PC1) are characterized by having high values of descriptors placed on the left side of the loading plot (negative values of loadings on PC1) and vice versa. The plot shows regions of both high and low variance as denoted by the two ellipses. The outer ellipse indicates 100% explained variance while the inner ellipse indicates that only 50% variance in the data could be explained. The first two PCs have explained 36% variance and that the outer 8 descriptors are those with highest loading (weight) on these PCs. The loading plot was analyzed with respect to score plot and the characteristics related to the distribution of chemicals in the PCA score plot are described in the following paragraph.

The descriptor C-026 (atom centered fragments) and MATS-1v (Moran autocorrelation of lag 1 weighted by van der Waals volume) have high weight for all chemicals in red marked sector which was dominated by class-I chemicals. Figure 1 and Figure 2 describe the scaffold for atom centered fragment. As shown in Figure 1 and Figure 2, the chemicals in the green marked sectors represent those with a halogenated alkene moiety, and are predominantly class I chemicals. It has been observed that along PC-2 the acyclic halogenated alkenes are present followed by more complex halogenated structures, e.g., halogen substituted aromatic and heterocyclic structures. The descriptor nCl (Number of Chlorine atoms) has a high weight for chemicals shown in the green marked sectors. The descriptor SpPosA_B(p) (Normalized spectral positive sum from Burden matrix weighted by polarizability) has a high weight for chemicals at right most side along PC1. The brominated chemicals laying at right most side as compared to chlorinated chemicals as bromine has a higher polarizability than chlorine. The acids, esters and alcohols were found along PC-1. Descriptors GATS1p (Geary autocorrelation of lag 1 weighted by polarizability) and GATS1v (Geary autocorrelation of lag 1 weighted by van der Waals volume) have a high weight for chemicals in the black marked sectors where class III chemicals are dominant. The descriptor Mi (Mean first ionization potential (scaled on Carbon atom)) and descriptor nDB (number of Double Bonds) shows high weight for chemicals in the upper-left quadrant and explains the increase of unsaturation from the left to right direction along PC-1. The class III chemicals are dominant in this region. We have also noted that the presence of phosphate and sulphate functionality increases upward along PC2. This is due to the weight that descriptors B01[S-P] (Presence/absence of S–P at topological distance 1), B03[C-S] (Presence/absence of C–S at topological distance 3) and P-117 (X3-P = X (phosphate)).

## 4. Conclusions

The aim of this study was to develop a preliminary QSAR model for the prediction of acute toxicity. The Munro database was used as the basis for model calibration, as it provides a general coverage of chemical space with respect to physico-chemical properties. Our study constitutes the first attempt to classify the Munro database chemicals on the basis of LD_50_ data. Three classes were formed using a GHS protocol that divided the Munro database chemicals based upon particular LD_50_ value thresholds. The most relevant molecular descriptors were selected by means of the Genetic Algorithm and used to develop a *k*-NN classification model for the Munro database chemicals. The further PCA analysis confirmed the importance of the selected descriptors in the clustering of chemicals based on structural features as well as highlighted their potential use for establishing a GHS scheme for classifying Munro database chemicals. We believe that this QSAR-based model for predicting acute toxicity may reveal unique insights concerning factors underlying acute toxicity and should be of interest for use in the preliminary screening of substances. Ultimately, this approach may contribute to a reduction in the use of animals in toxicity studies.

## Supplementary Materials

Supplementary materials can be found at http://www.mdpi.com/1422-0067/15/10/18162/s1.

## Supplementary Files

Supplementary File 1
